# Experimental dataset on leaf-based extracted antidandruff shampoo derived from Aloe Vera, Ocimum Sanctum, and Withania Somnifera: Advancing Ethiopian local herbs via formulation

**DOI:** 10.1016/j.dib.2024.110937

**Published:** 2024-09-17

**Authors:** Tegen Dagnew Tessema, Yohannes Shetawn Wendante, Belay Teffera

**Affiliations:** a Faculty of Chemical and Food Engineering, Department of Chemical Engineering, Bahir Dar Institute of Technology, Bahir Dar University, PO Box 26, Bahir Dar, Ethiopia; b Department of Mechanical Engineering, Universitat Rovira i Virgili, Av. Països Catalans 26, 43007 Tarragona, Spain

**Keywords:** Malassezia furfur, Herbal extracts, FTIR, Formulation, Antidandruff, ANOVA

## Abstract

Dandruff is a common fungal infection caused by the fungus Malassezia furfur. It is a big issue of concern in the human skin health system, as it is characterized by chronic non-inflammatory scalp conditions. These conditions include excessive scaling, itching, and redness. The fungus Malassezia is responsible for causing dandruff. Hence, this research dataset contains data related to the extraction and formulation of shampoo that cures dandruff by using extracts from the herbal leaves of Aloe Vera, Ocimum Sanctum, and Withania Somnifera herbs as an advancement of Ethiopian local herbs via formulation. The presented data provides an information on the optimal formulation process of herbal-based antidandruff shampoo against ketoconazole (KTC) as a control of the experiment. Ethanol-extracted oil from Aloe Vera, Ocimum Sanctum, and Withania Somnifera leaves was used to create three different formulation codes of F1, F2, and F3. The formulated antidandruff shampoo was evaluated in terms of its solid content, foamability, viscosity, dirt dispersion, pH, wetting time, and organoleptic properties. Moreover, the dataset includes information on the antifungal activity of the antidandruff shampoo against Malassezia furfur, as determined by disc diffusion and colony count testing method. The active chemical components responsible for the antidandruff activity of the leaf-extracted oil were also measured using FTIR.

Specifications Table

**Table:** 

Subject	Chemical Engineering
Specific subject area	Chemical Engineering, process intensification, and Pharmaceutical science
Data format	Raw
Type of data	Tables, Figures, Image(word, sigma plot, and excel)
Data collection	Healthy,well matured Aloe Vera (eret), Ocimum Sanctum (Damkese), and Withania Somnifera (gizewa) (Figure 1) were collected from northern part of Bahir Dar city Bezawit Mountain, Emperor Hailsilase Palace, Ethiopia in March 2023. The respective herbal leaves, were cleaned and then allowed to dried with air for one day at 45 °C using oven (M40-VF,MPM-instruments Italy -Oven Incubator). The leaves were subsequently grounded to a powder using a mortar and pestle to make ready for the next experiment. The equipment used for this experimental study were: ASoxhlet apparatus for the extraction of the oil from herbal leaves. A a sieve, an electronic weighing balance (CAS-164, 0.001 g) to measure the size and the weight of the raw materials and solid chemicals used in the formulation respectively. A pH meter was used to measure the acidity and basicity of the formulated antidandruff shampoo during antifungal performance evaluation. A drying oven (M40-VF, MPM-instruments Italy - Oven Incubator) was used to remove the moisture and solid content of the herbal leaves in the shampoo. A viscometer (VSCOSTAR+*H* FUNGILAB CO. Ltd, Barcelona) to measure the viscosity of the formulated antidandruff shampoo. A mortar and pestle (Fritsch D-55745idar, Oberstein, Germany) were used to grind the leaves into a suitable size for the extraction process. A digital photo calorimeter (Rs700-India), was used for the color measurement. Moreover, a JASCO FT/IR-6600 FT-IR spectrometer was used to record the infrared (IR) spectrum of extracts obtained from Aloe Vera, Withania Somnifera, and Ocimum Sanctum. The functional groups were then determined using IR correlation charts. The statistical method of central composite design and analysis of variance was used to test the significance of each experimental factors on the responses. Three experimental variables (time, volume ratio, and speed of mixing (RPM)) were chosen for the study. Volume ratio (75,50,25) in mL, mixing time(2,4,6)in hrs and mixing speed (2.5,5,10) in rpm with three levels. The total number of the experimental runs were (3 factors, 3 levels and 2 replications). After the successful extracting of the oil from the herbal leaves, and the formulating the antidandruff shampoo,we evalutaed its efficacy via various parameters including foam stability, Ph, viscosity, and antifungal activity. The antifungal activity was test was evaluated using zone of inhibition and the colony enumeration test specificaly disc diffusion test. The number of colonies was calculated using Eq. (1). To determine the functional group present,we used a JASCO FT/IR-6600 FT-IR spectrometer to record the infrared (IR) spectrum of extracts obtained from Aloe vera, Withania Somnifera, and Ocimum Sanctum, and correleted the results with IR correlation charts. The foaming ability and foam stability were determined using the cylinder shake method. In-vitro evaluations of anti-dandruff activity and antifungal activity were conducted using the agar diffusion method. The test organism
	was cultured in Sabouraud Dextrose Broth (SDB) with olive oil for 24 h, and the resulting broth culture was used to inoculate the tubes with the test ingredients. The tubes were then incubated at 30 °C for 24 h, following the procedures reported in. The plate count of the strain of Malassezia furfur was performed using the disc diffusion method. A 0.1 ml aliquot of the serially diluted soil sample was transferred onto SDB media and spread with a sterile spreader. The average value of the triplicate plates was then calculated to determine the colony forming units (cfu) of Malassezia. The number of colony forming units (CFU) was evaluated using the formula (1). The results of this test were classified as either acceptable or non-acceptable. Clinical isolates of fungal strain of, Malassezia furfur was collected from Microbiogy laboratory, Bahir Dar Institute of Technology, Bahir Dar University, Ethiopia. The strain was grown on Sabouraud Dextrose Broth (SDB) with olive oil for inoculation of the tubes with the test ingredients and incubated at 30 °C for 24 h.
Data source location	Institution: Bahir Dar Institute of Technology, Bahir Dar University, Bahir Dar Ethiopia GPS Coordinate (Latitude and Longitude):11.37°N&37.1°E
Data accessibility Related research article	Repository name: Mendeley data Data identification number:DOI:10.17632/vm963zghnw.1 Direct URL to data: https://data.mendeley.com/datasets/vm963zghnw/1

## 1 Value of the Data

• This research data set covers different antidandruff shampoo process formulation parameters that could be valuable for engineers and scholars who are keen to study and optimize the variables in the formulation process of antidandruff shampoo. This research data set can provide valuable insights for engineers and scholars who are interested in understanding the impact of various formulation parameters on the efficacy of antidandruff shampoo. These variables could be used as a base line for an extensive study of optimistic operating conditions in the development of antidandruff shampoo in the modern medical eco-system.
• The experimental data set contains the antifungal activity test which was acquired from disc diffusion method and the values were reported as the diameter of zone of inhibition. The results could not be converted a minimum inhibitory concentration. This enables, researchers to re experiment other more robust controlled antifungal/antibacterial testing protocols like Matrix-Assisted Laser Desorption/Ionization Time-of-Flight.
• The experimental data demonstrates the effect of formulation ratio, mixing time and mixing speed on the antifungal activity for Malassezia of the leaf based extracted herbs of Aloe Vera, Ocimum Sanctum, and Withania Somnifera.
• The experimental data reveals that this approach could be a fundamental data resources in the future for researchers and policy makers who are interested in herbal based medicine development for dandruff curing.

## 2 Background

Though different data sets were available in the literature with regard to the clinical and antimicrobial efficiency evaluation test for Aloe Vera, Ocimum Sanctum, and Withania Somnifera, there appears to be a limited number of datasets that are entirely focused on applications of different herbs for clinical purpose of dandruff curing via formulation. Among the different datasets which was reported in this topic,: it has been reported that Ocimum sanctum essential oil and its active exhibit antifungal activity by disrupting ergosterol biosynthesis and membrane integrity. Additionally the antifungal activity of Aloe vera pulp and its liquid fraction against plant pathogenic fungi has been observed in vitro test. Furthermore the crude extracts of Withania Somnifera and Cenchrus setigerus have been demonstrated in *in-vitro* level [[1], [2], [3]].However, there is a limited data on the synergistic performance of these herbal extracts for curing the application of specific antimicrobial strains is limited due to the low percentage of active phytochemical components present in the oil. Moreover, each these herbs possess exceptional phytochemical components one from the other in the view point of dandruff treatment. For example, Withania Somnifera, has antioxidant and anti-inflammatory properties that can promote a healthy scalp and hair. On the other hand, Ocimum Sanctum has antimicrobial and anti-inflammatory properties. Aloe Vera (olive)gives a complement as a natural moisturizer by hydrating the scalp and preventing excessive dryness, which can contribute to the formation of dandruff flakes.

Hence, the objective of this data set was to present the synergistic performance of three herbal extracts for the treatment of specific antimicrobial strains(i.e. Malassezia furfur).

## 3 Data Description

### 3.1 Class description

The data set consists of six tables and five figures. The experimental data set contains data that describe the effect of formulation ratio, mixing time and mixing speed on the antifungal performance of the leaf based extracts obtained from three different herbs (Aloe Vera, Ocimum Sanctum, and Withania Somnifera).. Table 1 depicts physical appearance and pH of the formulated antidandruff shampoo at different formulation code of F_1_, F_2_ and F_3_. Table 2 depicts evaluation of foamability and foam stability value of the anti-dandruff shampoo. Table 3 illustrates evaluation of percentage of solid contents and dirt dispersion values obtained from the experiment. Table 4 describes the formulated antidandruff shampoo viscosity evaluation. Table 5, shows the wetting time and surface tension test. Table 6, shows the composition of fillers and formulation ratios of herbal leaves extracted oil use in the formulation process

**Table 1:** Experiment 1 Evaluation of physical appearance (texture), color, and pH.

Formulation code	Color	Texture	pH
F1	Dark brown	Smooth	5.51 ± 0.02
F2	Dark brown	Smooth	5.61 ± 0.07
F3	Dark brown	Smooth	5.81 ± 0.02

*Data presented mean ±SD

**Table 2:** Experiment 2 Evaluation of the anti-dandruff shampoo foamability and foam stability.

	Foam volume (ml)		
**Time (min)**	F1	F2	F3
5	149.4 ± 0.6	154.0 ± 0.64	156.4 ± 0.15
10	147 ± 0.45	149.2 ± 0.77	152.0 ± 0.65
15	144.3 ± 0.26	145.1 ± 0.7	149.1 ± 0.66

*Data presented mean ±SD

**Table 3:** Experiment 3 Evaluation of formulation for percent of solid contents and dirt dispersion.

Formulation Code	Percentage solid content (%)	Dirt dispersion
F1	22.15 ± 0.42	None
F2	24.43 ± 0.79	None
F3	25.30 ± 0.87	None

*Data presented mean ±SD

**Table 4:** Experiment 4 Formulated antidandruff shampoo viscosity evaluation.

	**Viscosity (cp)**		
**RPM**	F1	F2	F3
2.5	8682.67 ± 0.92	8287.15 ± 0.64	8867.87 ± 0.73
5	5834 ± 0.54	5827 ± 0.45	5828 ± 0.23
10	4864 ± 0.66	4640 ± 0.76	5130 ± 0.56

*Data presented mean ±SD

**Table 5:** Experiment 5 Wetting time and surface tension test.

	**Wetting time(**s**)**		
**RPM**	F1	F2	F3
2.5	167.5 ± 0.72	186.15 ± 0.15	192.27 ± 0.73
5	172.1 ± 0.35	189 ± 0.35	196 ± 0.33
10	173.5 ± 0.66	193.5 ± 0.6	198.2 ± 0.46

	**Surface tension(dynes/cm)**		
**RPM**	F1	F2	F3

2.5	54.2 ± 0.23	49.15 ± 0.25	38.5 ± 0.73
5	42.2 ± 0.44	45.52 ± 0.45	36.5 ± 0.23
10	41.5 ± 0.66	44.5 ± 0.76	35.25 ± 0.56

**Table 6:** Composition of fillers and formulation ratios of herbal leaves extracted oil used for the experiment.

S. No	Ingredients	F1	F2	F3	Reference/s
1	Aloe vera	50 mL	25 mL	25 mL	This study
2	Ocimum S.	25 mL	25 mL	50 mL	This study
3	Withania S.	25 mL	50 mL	25 mL	This study
4	SLS	12 g	12 g	12 g	[1,3,4].
5	Citric acid	0.5 g	0.5 g	0.5 g	[1]
6	NaCl	2.4 g	3 g	5 g	[1]
7	Formaldehyde	0.05 ml	0.05 ml	0.05 ml	[1]
8	EDTA	0.5 g	0.5 g	0.5 g	[1]
9	perfume	0.5 ml	0.5 ml	0.5 ml	[1,3]
10	Distilled water	90 ml	90 ml	90 ml	[1]
11	NaOH	0.06 g	0.06 g	0.06 g	[1]

**Fig. 1:**
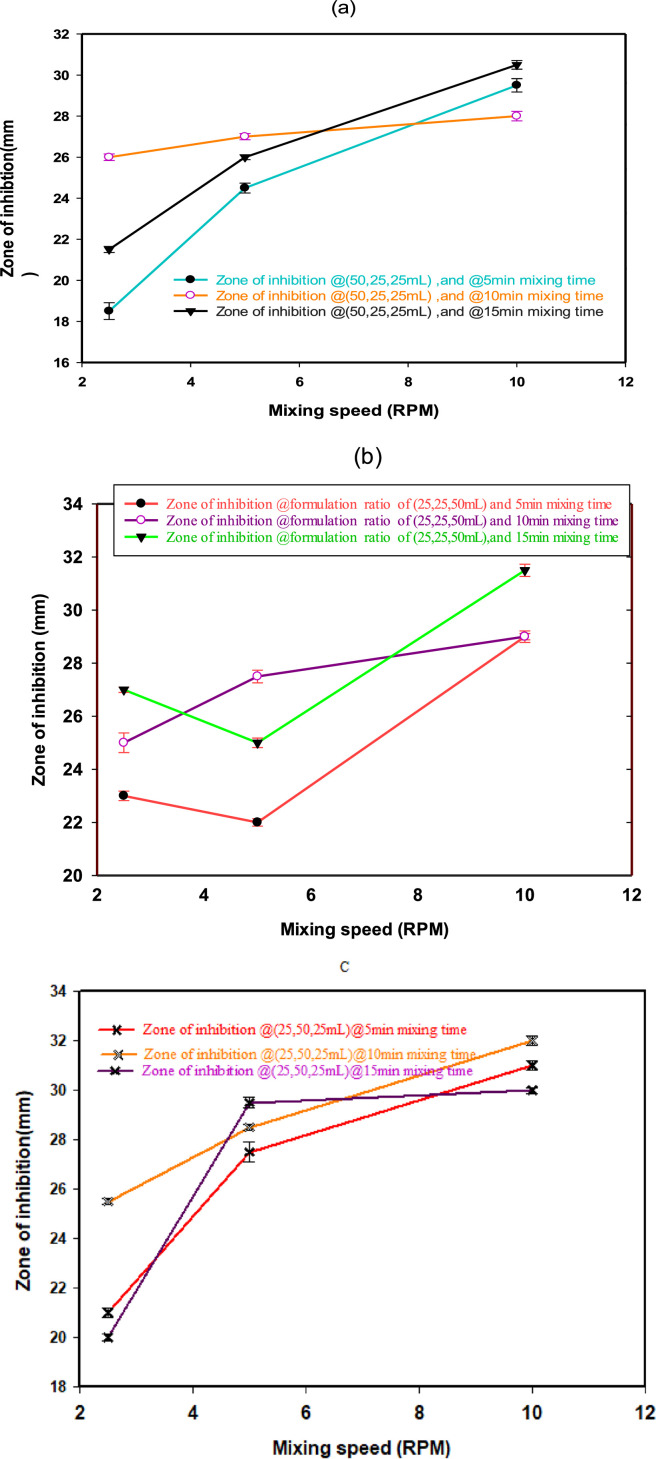
Experiment 6 - Anti-fungal activity of formulated antidandruff shampoo interms of zone of inhibition (mm): (a) for F1 (b) F2. (c) for F3 [provided as - DIB-D-24-01275.zip, /DOI:https://data.mendeley.com/datasets/vm963zghnw/1].

**Fig. 2:**
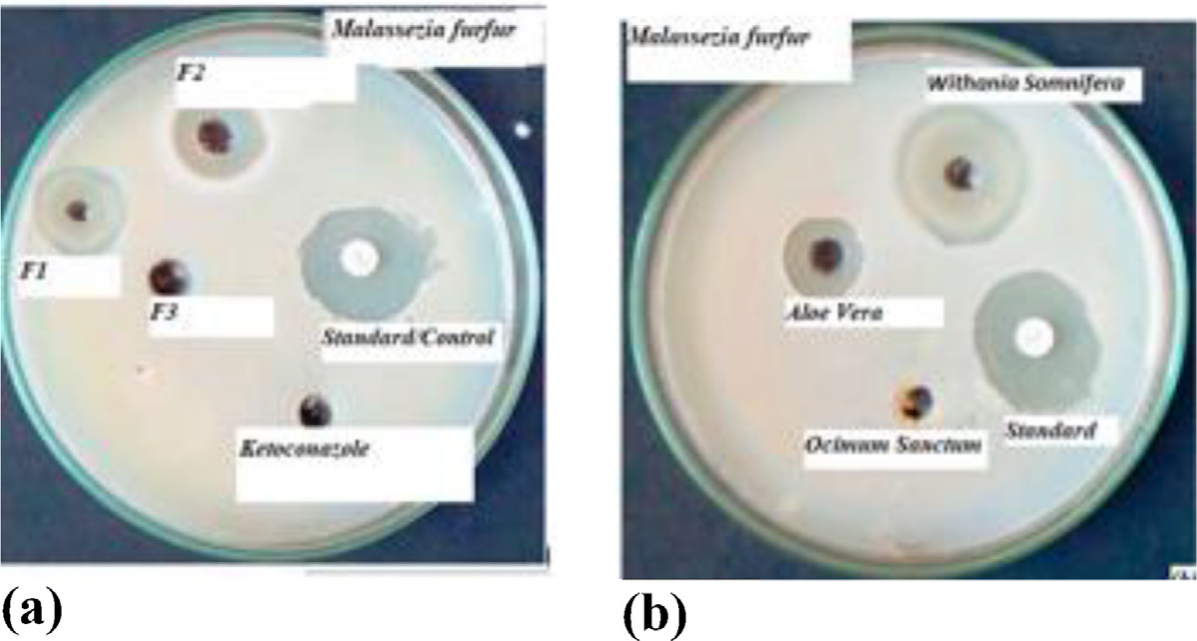
Experiment 7 - In Vitro test of zone of inhibition for Malassezia furfur (a) formulated antidandruff shampoos (b) Independent herbal antidandruff shampoos[provided as -DIB-D-24-01275.zip, /DOI:https://data.mendeley.com/datasets/vm963zghnw/1].

**Fig. 3:**
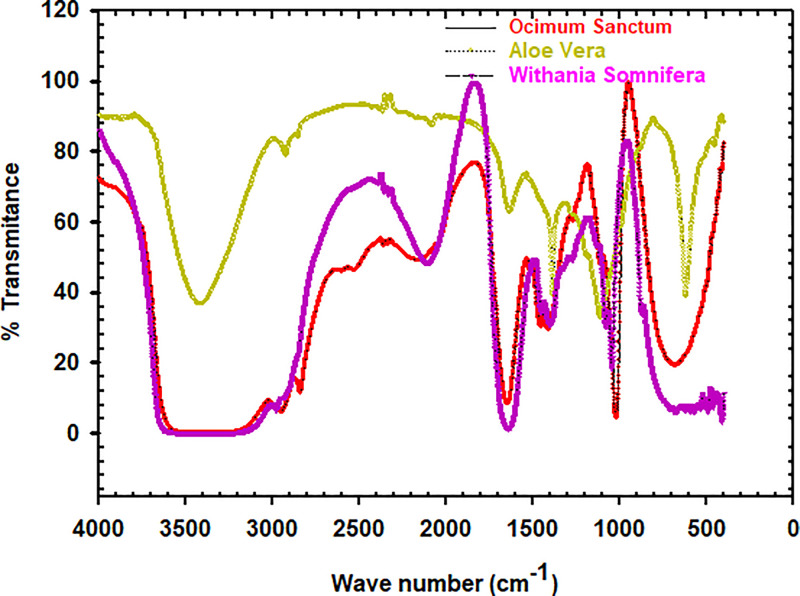
Experiment 8 - Fourier transform–infrared spectrum aided phytochemical components of the extracted oils of Ocimum Sanctum, Aloe Vera, and Withania Somnifera [Raw data´s provided as DIB-D-24-01275.zip, /DOI:https://data.mendeley.com/datasets/vm963zghnw/1].

**Fig. 4:**
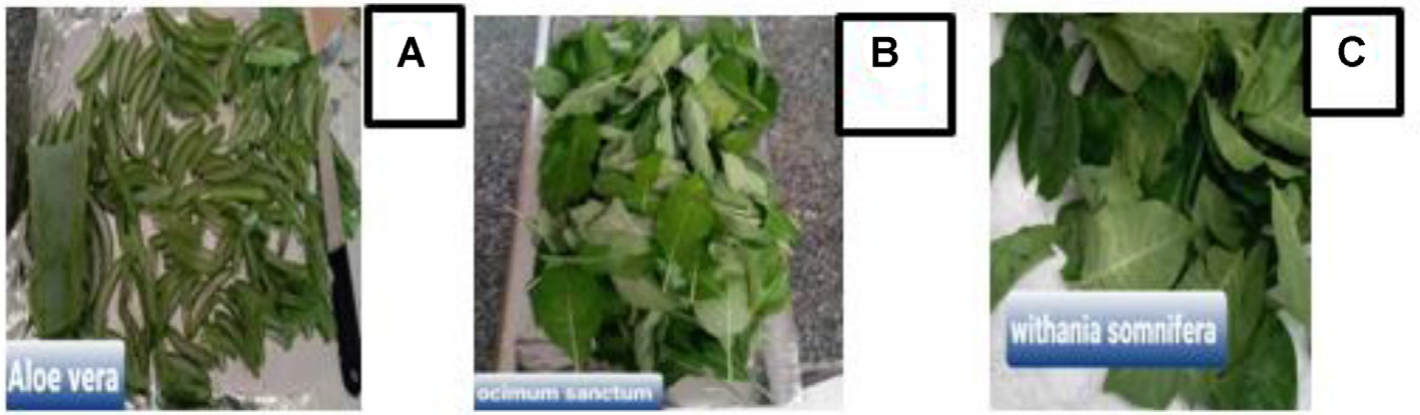
Herbal leaves used for the study **a)** botanical name***:Aloe Vera;*** common name**:(eret) b)** botanical name***; Ocimum Sanctum;*** common Name ***(damkese) c)*** botanical name***;Withania Somnifera,*** common name ***(gizewa*)**.

Fig. 1 illustrates the data for the anti-fungal activity of synthesized antidandruff shampoo in terms of zone of inhibition at a formulation codes of (a) for F1, (b) for F_2_ and (b) for F_3_. Fig. 2(a) shows in Vitro test for the zone of inhibition for Malassezia furfur and formulated antidandruff shampoos while (b) depicts the independent herbal antidandruff shampoos using disc diffusion method. Fig. 3, exhibits the active chemical components of FT-IR spectrum of Aloe Vera, Ocimum Sanctum, and Withania Somnifera extracts. Fig. 4. Illustrates herbal leaves with botanical name used for this study a) Aloe Vera b) Ocimum Sanctum c) Withania Somnifera.

**Fig. 5:**
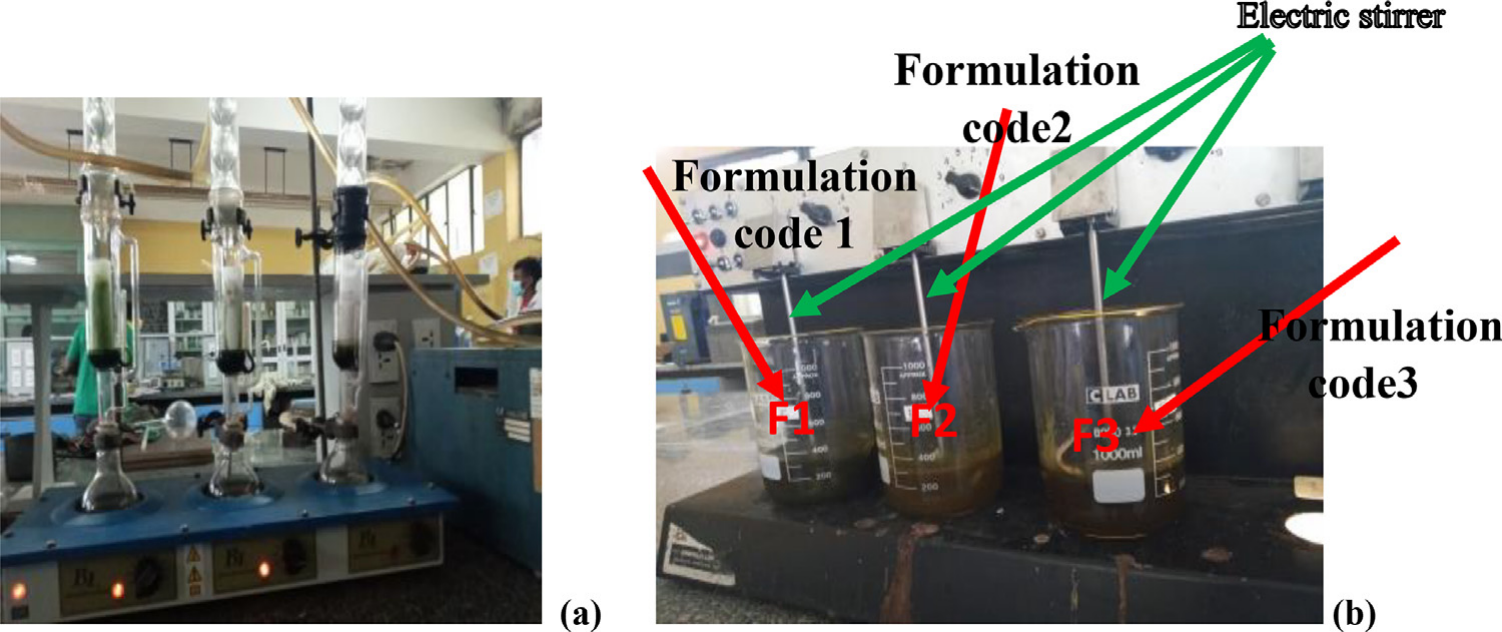
(a) Soxhlet extraction set up (b) Formulation of the antidandruff set up with mangetic stirrer.

Fig. 5 (a and b), depicts the experimental set up for the extraction process from the leaves of Aloe Vera, Ocimum Sanctum, and Withania Somnifer (a) Soxhlet extraction set up and (b) Formulation of the antidandruff set up with magnetic

### 3.2 Data set statistics

In this section we provide some general information of the data that provides the physico chemical quality parameters ` of the formulated antidandruff shampoo. The size of the data set deposited in Mendeley as a raw data (https://data.mendeley.com/datasets/vm963zghnw/1) with113kB.

### 3.3 Final data format and file structure

The entire data set is openly accessible in repository data folder. There are four data sets in the zip folder, three of them contains data related to the FTIR spectrum of Aloe Vera, Ocimum Sanctum, and Withania Somnifera. The fourth, it is an excel files which contains quality parameters and antifungal activity performance test results at different formulations of the antidandruff shampoo.

### 3.4 Classification of results

To evaluate the quality parameters of the antidandruff shampoo at different formulations, the following parameters were considered (Table 1, Table 2, Table 3, Table 4, Table 5), and Fig. 1, Fig. 2, Fig. 3, Fig. 4, Fig. 5. More detail methodologies, and experimental setup were described in Section 4.

Antidandruff shampoo, like any other cosmetic product, should have a pleasing physical appearance. Therefore, the formulated shampoos were evaluated its physical characteristics such as color The pH of a antidandruff shampoo is an important parameter for improving and enhancing hair quality, minimizing eye irritation, and maintaining the ecological balance of the scalp. The pH values of the formulated shampoos were measured and found to be in the range of 5.51–5.81, (Table 2). As result, the recommended pH value for antidandruff shampoo reported in the literature were between 5.5 and 7, which is in line with the current trend of promoting shampoos with lower pH to minimize hair damage, as reported elsewhere in [1,2].

## 4 Experimental Design, Materials and Methods

### 4.1 Experimental design

The statistical method of central composite design and analysis of variance was used to test the significance of each experimental factors on the responses as per the method employed in [5]. Three experimental variables (time, volume ratio, and speed of mixing (RPM)) were chosen for the study. Volume ratio (75,50,25) in mL, mixing time(2,4,6)in h and mixing speed (2.5,5,10) in rpm were selected with three levels. The total number of experimental runs was (3 factors, 3 levels and 2 replications) as illustrated in (Table 2).

The data presented in Fig. 2. was further analyzed using statistical software (SPSS IBMS 20) specifically through multivariate analysis of SPSS. The effect of formulation variables was evaluated using the ANOVA test in response to the antifungal activity. A *P* value less than 0.05 (*p* < 0.05) was employed to assess the interaction effect of factors which had a significant effect on the dependent variable (antifungal activity) and all the raw experimental data values were deposited provided as -DIB-D-24-01275.zip, /DOI:https://data.mendeley.com/datasets/vm963zghnw/1].

### 4.2 Materials, and methods (reagents, and data gathering process)

Sodium lauryl sulfate powder (C_2_H_25_O_4_S.Na),with a concentration of 70 % w/w (gms) was used as a detergent to facilitate the cleaning process. EDTA (C10H16N2Na2O8) powder with the purity of 99.8 % powder served as a chelating and sequestering agent. Ethanol (97 % w/v) with a concentration of 87 % W/V,was employed as a clarifing agent to extract the oils from the leaves used for extracting the leafʼs oil as a clarifying agent in litter. Formaldehyde liquid with a concentration range of 37–41 % w/v was used as a preservative in the amount of a one liter. Sodium hydroxide pellets with a purity of 98 % w/w were emplyoyed for neutralization,while citric acid anhydrous with a purity of (99.7 %) was used for PH adjustment, Sodium chloride powder with 98.5 % purtity w/w was utilized as a thickening agent. Distilled water was used for dilution and to measure surface tension. All chemicals and reagents used in this study were of analytical grades and purchased from LBS Marg, Mumbai - 400,086, India.

#### 4.2.1 Equipments /apparatus/ used

The equipment used for this experimental study were: A Soxhlet apparatus for the extraction of the oil from herbal leaves. A sieve, an electronic weighing balance (CAS-164, 0.001 g) to measure the size and the weight of the raw materials and solid chemicals used for the formulation respectively. A pH meter was used to measure the acidity and basicity of the formulated antidandruff shampoo during antifungal performance evaluation. A drying oven (M40-VF, MPM-instruments Italy - Oven Incubator) was used to remove the moisture and determine the solid content of the herbal leaves in the shampoo. A viscometer (VSCOSTAR+*H* FUNGILAB CO. Ltd, Barcelona) to measure the viscosity of the formulated antidandruff shampoo. A mortar and pestle (Fritsch D-55745idar, Oberstein, Germany) were used to grind the leaves to a suitable size for the extraction process. A digital photo calorimeter Rs700-India,was used for the color measurement. Moreover, a JASCO FT/IR-6600 FT-IR spectrometer was used to measure the infrared (IR) spectrum of extracts obtained from Aloe Vera, Withania Somnifera, and Ocimum Sanctum, based on the functional groups using IR correlation charts.

#### 4.2.2 Collection of plant materials and preparation for extraction

Healthy, well matured Aloe Vera (eret), Ocimum Sanctum (Damkese), and Withania Somnifera (gizewa) (Fig. 3) were collected from north-east part of Bahir Dar City, Bezawit Mountain Emperor Hailsilase Palace, Ethiopia in March 2023. Then, the herbal leaves, were cleaned and allowed to dry with air for one day at 45 °C using oven (M40-VF,MPM-instruments Italy -Oven Incubator). The leaves were subsequently grounded to a powder using a mortar and pestle to make ready for the next experiment.

Fresh and healthy leaves of (Aloe Vera, Ocimum Sanctum (damkese), and Withania Somnifera (gizewa)) were dried in the oven at 80 °C for 48 h, and then twenty grams of each powder were soaked in 200 ml distilled water for 24 h. The oil was extracted using 80 % ethanol at room temperature via the aqueous alcohol extraction as per the method employed in [4]. The crude extracts were then filtered through Whatman filter paper, and the filtrate was evaporated using a rotary evaporator under low pressure adopted from somewhere else [6,7]. The dried extract was further powdered and then dissolved in distilled water. The extracts were allowed to transferred to the glass vial and kept at 4 °C for the next experiments.

#### 4.2.3 Formulation of the herbal antidandruff shampoo

Sodium lauryl sulfate 12 g (12 %) was dissolved in distilled water, and five grams of citric acid was added into the beaker containing solution of sodium lauryl sulfate followed by addition of five grams of viscosity modifiers. Sodium Chloride was added to adjust the viscosity of shampoos and 0.5 mL of formaldehyde was added as preservative. The pH of shampoo was adjusted using 0.6 g of sodium hydroxide followed by the addition of 5 mL perfumes 0.0.5 % red dye, and (25–50 %) of herbal extracts i.e. Aloe Vera (local name, eret), Ocimum Sanctum (local name, damkese), and Withania Somnifera (local name, gizewa) in different formulation ratios as indicated in (Table 6).

#### 4.2.4 Strains and media preparation

Clinical isolates of the fungal strain of, Malassezia furfur was collected from the Microbiogy laboratory at Bahir Dar Institute of Technology, Bahir Dar University, Ethiopia. The strain was grown in Sabouraud Dextrose Broth (SDB) with olive oil for inoculation of the tubes with the test ingredients and incubated at 30 °C for 24 h as per the procedure reported in [8,9].

#### 4.2.5 Quality parameter evaluation of the anti-dandruff herbal shampoo

*Organoleptic evaluations*: The formulated herbal anti-dandruff shampoos were evaluated for their organoleptic properties in terms of physical appearance (i.e. color), and texture. [10] The values of texture were reported after having the fifteen trained panelists evaluation of the antidandruff shampoo and the results were presented as mean followed by standard deviations (Table 2) [8]. The panelist score ranges from 1 to 3, 1 indicates rough,2 indicates null, and 3 indicates, smooth (Raw data´s provided as -DIB-D-24-01275.zip, /DOI:https://data.mendeley.com/datasets/vm963zghnw/1]). Moreover, the physical appearance of the antidandruff shampoo was measured in terms of color using a digital photo calorimeter as per the method reported in [8,11]**.**

#### 4.2.6 Evaluation of physico-chemical parameters for the formulated antidandruff shampoo

**pH**: A 10 % v/v shampoo solution diluted in distilled water and the pH of the solution was measured by using a calibrated pH meter as per the method employed in [12,11].

*Percentage solid content:* A clean, dry evaporating dish was weighed and four grams of shampoo were added to the evaporating dish. The dish and shampoo were weighed, and then the exact weight of the shampoo was calculated. The evaporating dish with shampoo was placed on the hot plate until the liquid portion was evaporated. The weight of the shampoo (only solids) after drying was calculated as per the method reported in [13].

*Dirt dispersion***:** Two drops of herbal shampoo were added in a large test tube containing 10 mL of distilled water with one drop of India ink. Then, the test tube was stoppered and shaken ten times. Afterwards, the amount of ink in the foam was estimated as none, light, moderate, or heavy, as per the method employed somewhere else [14].

*Foaming ability and foam stability:* The cylinder shake method was employed for determination of foaming ability. A 50 ml solution of 1 % shampoo was poured into a 250 mL graduated cylinder and the cylinder was covered with aluminum foil and it was then shaken for 10 times. The total volumes of the foam contents after 1 min of shaking were recorded. The foam volume was calculated immediately after shaking and a 1 min interval for four minutes. Foamability was recorded the volume of foam at 1 min intervals for 4 min and foam stability were recorded according to the method reported somewhere [15].

*Wetting time test*: The wetting ability of the surfactant was tested via a small piece of paper cut in to small pieces with a diameter of 1 in. and a mean weight of 0.44 g. The disc was placed on the surface of 1 % v/v formulated shampoo solution and the time was recorded with stop watch. Afterwards, the time required for the disc to begin sinking was noted as the wetting time of the formulated shampoo solution based on the method reported in somewhere else [[16], [17]].

*Surface tension measurement:* was performed out with 10 % w/v formulated shampoo in a distilled water using stalagmometer calibrating at room temperature [18].

*Rheological Property evaluations:* The viscosity profile of the formulated shampoos was determined by using VISCO STAR PLUS viscometer set at different spindle speeds ranging from 0.3 to 10 rpm. The viscosity of the shampoos was measured by using spindle R4 (5 rpm). The temperature and sample container's size was kept constants during the study [19].

*In-vitro anti-dandruff activity evaluations:* Antifungal activity tests were performed using the agar diffusion method with a twenty-four hour broth culture of the test organism and the culture in the Sabouraud Dextrose Broth (SDB) with olive oil for inoculation of the tubes with the test ingredients. The tubes were then incubated at 30 °C for 24 h as per the work reported in [20].

Afterwards, a loop full of broth culture was streaked on an SDB plate over laid with olive oil to detect the presence or absence of growth of Malassezia (MTCC: 1374). Antidandruff shampoo (4 μl) (containing active ingredients) was weighed separately in the Petri plates and the molten SDB was poured and mixed thoroughly followed by culture (20 μL) to spread over in the agar media. The plates were incubated at 30 °C for 3–5 days, following the method reported in [21]. All experiments were carried out in triplicate using Ketoconazole as a control. Fungal culture (e.g., Malassezia furfur), agar plates (e.g., sabouraud agar), sterile pipettes, an incubator, antifungal agents (test samples), sterile swabs, sterile distilled water or saline solution, sterile loops or spreaders, and petri dishes were used for colony enumeration, and the zone of inhibition test [22].

The plate count of the strain of Malassezia furfur was carried out using a disc diffusion method. This involved transferring 0.1 ml of the serialy diluted soil sample on to SDB media and spread it with sterile spreader. The average value of the triplicate plates was taken to determine the number of colonies expressed as a colony forming units (cfu) of Malassezia. The number of colony forming units (CFU) was evaluated using Equ^n^(1) Raw data´s provided as -DIB-D-24-01275.zip, /DOI: https://data.mendeley.com/datasets/vm963zghnw/1].

The test results were evaluated as either acceptable or non-acceptable score. The standard range for bacterial count on the plate was determined to be 250-300CFU as reported elsewhere [22].

Colony forming units (Cfu)

(1) $$=\;\frac{\text{average}\;\text{number}\;\text{of}\;\text{colony}\;\text{for}\;\text{triplicate}}{ammount\;of\;sample\;transfered\;to\;the\;media}\;*\text{inverse}\;\text{dilution}\;\text{factor}$$

#### 4.2.7 Functional properties and structural information of the herbal leaf extracts

JASCO FT/IR -6600 FT-IR spectrometer was used to characterize the functional groups based on the chemical structures. The FT-IR spectra of Aloe Vera, Withania Somnifera, and Ocimum sanctum oil was acquired using a Jasco spectrum 6600 FT-IR spectrometer, and functional groups were determined using IR correlation charts.

*SPSS tools used for analysis:* The experimental data were statistically evaluated using IBMS 20 statistical software. A univariate and one-way analysis of variance (ANOVA) were employed at level of significance (cut of point≤ 0.05) to detect differences among the treatment of means.

## Limitations

There was some limitations while producing this research datasets. The antifungal performance test of Malassezia Furfur was evaluated using disc diffusion method and the results were reported as the diameter of zone of inhibition which could not be directly converted to a minimum inhibitory concentration. As a result we strongly recommend future users of this data to use it as a base line to see the discrepancy and compare with other more robust controlled antifungal/antibacterial testing protocols such as Matrix-Assisted Laser Desorption/Ionization Time-of-Flight.

In addition, during the organoleptic evaluations, there were variations in the scores given by the panelists for texture evaluation. Thus, conducting experiments on properties of the antidandruff shampoo based on respondent indices, such as texture, was a challenging task. The variations were reported using normalized values of the mean followed by standard deviation. Hence, for future use of this data for parameters that require panelists testing, the experimentalist ensure that the panelists are well-trained, undergo regular audits, normalization, feedback loops, and standardization. This will help to obtain a consistent and reliable dataset for the parameters being studied.

## Ethics Statement

This study does not involve studies with animals or humans. Therefore, we confirm that our research strictly adheres to the guidelines for authors provided by Data in Brief in terms of ethical considerations.

## CRediT Author Statement

**Tegen Dagnew Tessema:** Supervised, designed the experiments (conceptualization), collected and analyzed the data, wrote the original draft, reviewing and Editing. **Yohannes Shitahun Wondante:** Designed the experiments (conceptualization), collected and analyzed the data. **Belay Teffera Yalew:** Designed the experiment and reviewing**.**

## Data Availability

Data set__Antidandruf formulation from Aloe vera_ Ocimum sanctum_ and Withania Somnifera (Original data) (Mendeley Data)

## References

[1] Khan A. (2010). Ocimum sanctum essential oil and its active principles exert their antifungal activity by disrupting ergosterol biosynthesis and membrane integrity. Res. Microbiol..

[2] Jasso De Rodríguez D., Hernández-Castillo D., Rodríguez-García R., Angulo-Sánchez J.L. (2005). Antifungal activity in vitro of Aloe vera pulp and liquid fraction against plant pathogenic fungi. Ind. Crops Prod..

[3] Singariya P., Mourya K.K., Kumar P. (2012). Antimicrobial activity of the crude extracts of withania somnifera and Cenchrus setigerus In-vitro. Pharmacogn. J..

[4] Singariya P., Mourya K.K., Kumar P. (2012). Antimicrobial activity of the crude extracts of withania somnifera and cenchrus setigerus in-vitro. Pharmacogn. J..

[5] Tessema T.D., Yemata T.A. (2022). Power Production using a Batch Double-Chamber Microbial Fuel Cell from Brewery Wastewater: Effects of Electron Acceptors. Int. J. Energy Res..

[6] Reddy V.S., Gopinath C. (2018). Formulation and evaluation of synthetic anti-dandruff shampoo. Asian J. Pharm..

[7] R.K. Jaggi, R. Madaan, and B. Singh, “Anticonvulsant potential of holy basil, Ocimum sanctum Linn., and its cultures,” 2003. 15332507

[8] Pankhurst R. (2001). Proceedings of the National Workshop on biodiversity conservation and sustainable use of medicinal plants in Ethiopia.

[9] Lim D. (2023). Clinical efficacy of a gentle anti-dandruff itch-relieving shampoo formulation. Int. J. Cosmet Sci..

[10] Handa S.S. (2008).

[11] Bagatharia S.B., Thaker V.S. (2005). Antibacterial activity of Aloe vera leaf gel extracts against Staphylococcus aureus. Indian J. Microbil..

[12] Shi X.D., Yin J.Y., Huang X.J., Que Z.Q., Nie S.P. (2018). Structural and conformational characterization of linear O-acetyl-glucomannan purified from gel of Aloe barbadensis Miller. Int. J. Biol. Macromol..

[13] Al Badi K., Khan S.A. (2014). Formulation, evaluation and comparison of the herbal shampoo with the commercial shampoos. Beni-Suef Univ. J. Basic Appl.Sci..

[14] Malvia D., Sharma R.K. (2014). Advancement in shampoo (a dermal care product): preparation methods, patents and commercial utility. Recent Patents Inflamm. Allergy Drug Discov..

[15] Golhani D., Pandey V., Shukla A., Shukla R. (2015). Formulation and comparative evaluation of herbal shampoo with marketed products. Mintage J. Pharm. Med. Sci..

[16] Pawar A.P., Pawar D.N., Dalvi Y.V. (2019). Formulation and evaluation of polyherbal soap. Res. J. Top. Cosmet. Sci..

[17] Heidari B.A., Badinloo S., Ohadi M., Noudeh G.D. (2016). Bioencapsulation of biosurfactant-producing Bacillus subtilis (PTCC 1023) in alginate beads. Jundishapur J. Nat. Pharm. Prod. 2016 11:4.

[18] Arora R., Singh R.K., Meenakshi B. (2019). PCJHBA formulation and evaluation of herbal shampoo by extract of some plants. Pharm. Chem. J..

[19] Mainkar A.R., Jolly C.I. (2000). Evaluation of commercial herbal shampoos. Int. J. Cosmet. Sci..

[20] Matuschek E., Brown D.F.J., Kahlmeter G. (2014). Development of the EUCAST disk diffusion antimicrobial susceptibility testing method and its implementation in routine microbiology laboratories. Clin. Microbiol. Infect..

[21] Gaud R.S., Gupta G.D. (2001).

[22] R U., S D., P I. (2017). Preparation of herbal shampoo (HS) by green method and their characterization. IJRSSIS.

